# Do longer consultations improve the management of psychological problems in general practice? A systematic literature review

**DOI:** 10.1186/1472-6963-7-71

**Published:** 2007-05-17

**Authors:** Catherine Hutton, Jane Gunn

**Affiliations:** 1 General Practice, Moonee Ponds, Victoria, 3039, Australia; 2 Primary Care Research Unit, Department of General Practice, The University of Melbourne, Berkeley St, Carlton, Victoria, 3053, Australia

## Abstract

**Background:**

Psychological problems present a huge burden of illness in our community and GPs are the main providers of care. There is evidence that longer consultations in general practice are associated with improved quality of care; but this needs to be balanced against the fact that doctor time is a limited resource and longer consultations may lead to reduced access to health care.

The aim of this research was to conduct a systematic literature review to determine whether management of psychological problems in general practice is associated with an increased consultation length and to explore whether longer consultations are associated with better health outcomes for patients with psychological problems.

**Methods:**

A search was conducted on Medline (Ovid) databases up to7 June 2006. The following search terms, were used:

general practice or primary health care (free text) or family practice (MeSH)

AND consultation length or duration (free text) or time factors (MeSH)

AND depression or psychological problems or depressed (free text).

A similar search was done in Web of Science, Pubmed, Google Scholar, and Cochrane Library and no other papers were found.

Studies were included if they contained data comparing consultation length and management or detection of psychological problems in a general practice or primary health care setting. The studies were read and categories developed to enable systematic data extraction and synthesis.

**Results:**

29 papers met the inclusion criteria. Consultations with a recorded diagnosis of a psychological problem were reported to be longer than those with no recorded psychological diagnosis. It is not clear if this is related to the extra time or the consultation style. GPs reported that time pressure is a major barrier to treating depression. There was some evidence that increased consultation length is associated with more accurate diagnosis of psychological problems.

**Conclusion:**

Further research is needed to elucidate the factors in longer consultations that are associated with greater detection of psychological problems, and to determine the association between the detection of psychological problems and the attitude, gender, age or training of the GP and the age, gender and socioeconomic status of the patient. These are important considerations if general practice is to deal more effectively with people with psychological problems.

## Background

The consultation is a crucial component of general practice. It is how general practitioners (GPs) organise their days, is often related to how they get paid, and is used to assess the quality and impact of their practice. For at least three decades there has been discussion on how to achieve what has been described as "the exceptional potential of each consultation [1]". This is driven in part by the concern that time pressure is a limiting factor in the standard of general practice care provided [2-5].

Psychological problems present a huge burden of illness in our community, depression being listed as the fourth most prevalent chronic illness [6]. GPs are the main providers of care to those with psychological problems [7]. In Australia, between 2000 and 2002, psychological problems were managed at a rate of 11.5/100 general practice encounters [8]. But in the United Kingdom up to 40% of patients presenting to general practice are psychologically distressed as measured by screening tools, such as the General Health Questionnaire [9]. So it is possible that the patients recognised by GPs may be a small percentage of the total number of those suffering from mental illness [10]. Some believe that only about half of patients with psychological problems will be recognised if they do attend general practitioners [11].

Alongside these findings are workforce issues and GP shortages. Australia [12], UK [13], USA [14,15] and Canada [16] face medical workforce shortages which are compounded by changing demographics, including feminization, and doctors working less hours [17]. China [18] and many African [19] countries also have severe workforce shortages that are worsened by the emigration of physicians to developed countries [20].

Doctor time is an important resource for our community. Doctors spend time "*in face-to-face contact with patients..., doing administrative work related to visits, and maintaining a knowledge base. Importantly, time is always finite: no mater what demands*" a doctor " *faces, there are only 24 hours a day*"[21]. Time management for GPs has become a crucial issue. There is increasing evidence that links longer consultations with improved quality of care [5,22-24] but there is appropriate concern that longer consultations will lead to reduced access to GPs and the inability for patients to be seen in a timely manner

Of course, available evidence on the benefits of longer consultations has to be weighed against the provision of acute services to the community and patients' needs and expectations.

### Previous literature reviews

Prior to commencing this review we identified five previous relevant literature reviews published between 1992 and 2002 that focussed on factors associated with consultation length in general practice. However, none included clinical outcome measures, randomised controlled trials or specifically explored the management of common psychological problems, such as depression.

Wilson et al [2] reviewed 42 papers to examine "historical and international comparisons of consultation length" and discussed factors that determine consultation length concluding that the mean consultation length in UK had increased, and that "doctor factors" determined the length of consultation rather than differences between patients.

Groenewegen and Hutten [21] in the Netherlands reviewed the literature on "determinants and consequences of workload and job satisfaction of GPs" and concluded that the length of consultation was an important determinant for "workstyle" and quality of work. They found that the length of consultation was determined by both patient-initiated demand and by management decisions of the GP.

In the USA, Dugdale et al [22] explored the effects of limiting time on the doctor-patient relationship and concluded that the visit rate of greater than 3 or 4 patients/hour may lead to "suboptimal visit content, including decreased patient satisfaction and increased prescribing."

Freeman et al [5] reviewed outcomes of the debate about length of consultation and reported longer consultations were associated with a "*range of better patient outcomes, particularly better recognition and handling of psychological problems"*, and recommended that longer consultations should be a priority.

Recently, Wilson and Childs [23] published a systematic literature review of thirteen papers in which they explored associations between consultation length and consultation process and "process and healthcare" outcomes. They concluded that the evidence suggests that a patient *"seeking help from a doctor who spends more time with them is more likely to have a consultation that includes important elements of care."*

We set out to gather together and review the published literature exploring the associations between consultation length and the management of psychological problems.

## Methods

A search was conducted for English language publications on Medline (Ovid) databases up to 7 June 2006. A similar search was done in Web of Science, Pubmed, Google Scholar, and Cochrane Library and no other papers were found. The following search terms, including Medical Subject Headings (MeSH) were used:

general practice or primary health care (free text) or family practice (MeSH)

AND consultation length or duration (free text) or time factors (MeSH)

AND depression or psychological problems or depressed(free text).

105 possible papers were found in this way.

Studies were included if they contained data on consultation length and factors related to the detection and management of psychosocial problems in a general practice or primary care setting. Eleven studies of the original 105 met the inclusion criteria. The reference lists of the 11 identified articles were checked and all additional relevant articles included. Other relevant papers by the included authors were retrieved and read. Finally, 29 possible articles were identified for inclusion in the review.

CH read and re-read the papers in conjunction with JG. The studies were then arranged into tables, listing the aims, methods, data and findings. This enabled data extraction in a systematic way. A thematic analysis was undertaken by CH and themes were refined after discussion with JG.

## Results

According to these inclusion criteria, 29 papers were eligible for this review. The majority of the studies were observational, cross-sectional studies. There were two intervention studies published in three papers. No meta-analysis of published studies and no relevant randomised controlled trials were identified

### Quality of included studies

The quality of studies varied widely and is discussed below providing examples. Further information is included in Tables 1, 2 and 4.

**Table 1:** Studies based on interviews of doctors about consultation length and the management of psychological problems

**Author/year/location**	**Aim**	**No. of practices/doctors**	**No of patients/consultation**	**Mean consult'n length**	**Method of measuring consult'n length**	**Method of study**	**% of eligible doctors particip'ing**	**Conclusions/findings**
Rost USA 1994 [28]	To describe preferences & barriers to rural primary care physicians treating depression		53			Semistructured Interviews	86% Random sample	30% of primary care physicians state that lack of time, & 23% that patient not recognising problem, is the biggest barrier to treating depression
Howe 1996 UK [44]	To assess factors that influence GPs' identification of psychological distress	-/19 GPs, random sample in Sheffield	-	-	-	GPs sent postal 'questionnaire, then semi-structured interviewed		Time shortage recorded as factor in 15/19
Pollock 2003 UK [31]	To investigate GP perspectives on consultation times and the management of depression in general practice	8/19 Not representative	-	8–10 mins booking times	-	Qualitative, cross-sectional GP semi-structured interviews		Dealing with depression, particularly first consultation, takes longer. GPs accommodate this by running over time.
Smith 2004 UK [32]	To explore GPs' views on clinical guidelines on management of depression & barriers to use	-/11. Picked to representative of GPs	-	5–10 minute booking interval	-	Qualitative, cross-sectional In-depth interviews with GPs	73%	Lack of time major barrier to guideline use

**Table 2:** Aims and methodology of cross-sectional studies on consultation length and the management of psychological problems.

**Author/year**	**Aim**	**Duration**	**No. of practice/doctors**	**No of patients/consult'n**	**Mean consult'n length**	**Method of measuring Consult'n length**	**Type and method of study**	**% of eligible participating**
Westcott UK 1977 [25]	To study length of general practice consultations and patient characteristics.	2 weeks	1/1-the author	182 patients	8.66 mins	Timed by doctor	Quantitative Data recorded by doctor	100% of all surgery consultation.
Raynes UK 1980 [38]	To determine which characteristics of GPs, patients & consultations contribute to differences in consultation length, esp. with psychosocial problems.		-/10	264	4.2–8.7	Recorded by non-participating observer	Quantitative Consultations observed and analysed, GP questionnaire	Only 4 patients refused. 1.5%
Hughes UK 1983 [3]	To assess whether length of booked appointments affected consultation outcomes	12 weeks	2/6	1652 consultations	Practice A: 8 min, 4 secs. Booked 10 m Practice B: 5 min 18 s. Booked 5 min	Timed over some sessions	Quantitative Doctor filled encounter forms	-Not stated
Whitehouse UK-Manchester 1987 [29]	To study factors that influence the management of psychosocial illness in general practice	-	-/201	6870 consultation with psychosocial diagnosis,	<6, 6–6.99, 7–7.99, >8	Recorded by doctor	Quantitative Doctor encounter forms	40%, representative sample
Andersson 1989 Sweden [37]	To test hypothesis that longer consultations provide greater satisfaction to the doctor & patient.	20–40 consec. Consult'ns	4/7 male doctors with interest in research	160 consultations	21 mins	Doctor recorded-from greeting to farewell	Quantitative Doctor and patient questionnaire	-Not stated
Howie UK-Lothian (Scotland 1991/1989 [24, 36]	To examine association between different consulting styles, consultation length & prescribing, between quality of consultation, working style of doctors and length of consultation, (slow, intermediate and fast).	1 year	-/85	21,707, 1787 for RTI	Fast <7 mins, Intermediate = 7–8.99, Slow >9 mins	Timed by doctor	Quantitative Doctor completed encounter forms, some patient questionnaires	17%
Andersson Sweden 1993 [52]	To study factors assoc with short and long consultations	80 consec. consult'ns	3/6-all male	80 each doctor	66 consultations <10 mins, 314 between 11–30 mins 83 >31 mins	Recorded by doctor	Quantitative Patient questionnaires GP questionnaires	96.4%
Winefield Australia 1996 [40]	To assess relation between patient-centredness (PC), patient satisfaction and consultation length.	Consecutive appointments	-/21	10 per doctor = 210	16.9 mins for high doctor PC, 10.6 mins for low	Audiotapes	Quantitative Consultations audio taped and analysed for PC	41% GPs 82.5% patients
Martin Australia 1997 [45]	To assess characteristics of longer billed consultations.	1984–1992	-/-	-	Longer consultations>20 or >25 mins Mean = 14.6 mins [54]	Medicare data. Aust Morbidity & Treatment survey ACT Record Linkage survey	Quantitative Retrospective analysis Data from government records	AMTS-50.4% ACT Record Linkage Survey- 94% [48]
Carr-Hill UK 1998 [39]	To study characteristics of patients, GPs and practices associated with variation in consultation length.	2 weeks	10/51	836	GP averages between 4.4–11.0 mins	time with patient, measured by research nurse who sat in	Quantitative Research nurse sat in consultation & recorded data.	Not stated
Blumenthal Boston USA 1999 [30]	To determine the patient, practice, physician and visit characteristics that affect consultation duration.		-/686	Random sample picked from 19,192	16.3 mins	Recorded by office staff	Data obtained from 1991–1992 National Ambulatory Medical Care Survey & physician interviews Encounter forms	72% of a random sample of doctors
Howie UK UK 1999 [26]	To study relationship between, patient enablement scores, consultation length & quality as measured from NHS data.	2 weeks	53/221	25994	8	Doctor timed	Quantitative Doctor encounter forms & survey, patient questionnaires	38%
Stirling UK, Glasgow 2001 [9]	To examine factors in GP associated with diagnosis & management of psychosocial distress and consultation length	6 months	9 (all accredited for training)/21	1075 consult'ns (about 50 each GP)	8.71 mins (SD = 4.4)	Timed by observer in waiting room	Quantitative Patients completed GHQ-12 and questionnaire, GP rated psychological distress	Not stated Not representative
Harman New York, USA 2001 [42]	To determine the factors in a doctor's visit associated with recognition of depression			17058 consult'ns	16.4 mins without depression, 19.3 mins with depression	Recorded by doctor	Quantitative Data from National Ambulatory Medical Care Survey, 1998 Encounter forms	67.9% of doctors, random sample
Deveugele Belgium, Spain, UK, Switzerland., Germany, Netherlands 2002 [43]	To explore the determinants of consultation length in general practice across six European countries	-	190	3674	G = 7.6, Sp = 7.8, UK = 9.4, N = 10.2, B = 15, SW = 15.6 mins	Measured by stopwatch	Quantitative Videotaped consultations	79% of patients
Telford 2002 UK [34]	To survey GPs' views on barriers to the provision of good management of depression	-	-/1703	-	-		Qualitative, cross-sectional GPs sent postal questionnaire	48%
Tahepold Estonia, 2003 [41]	To study influence of patients' age, gender & problem on length of consultation.		-/27	405	9.0 mins	Videotaped	Quantitative Videotaped consecutive consultations, analysed	98% of patients Doctors not stated
Britt Australia 2004 [17]	To examine relations between billed consultation length and content.	Apr 2000 – Mar 2002	-/2811	101112	<20 mins, >20 mins Mean = 14.6 mins [54]	Doctor recorded & Medicare data	Quantitative Doctor encounter forms & Medicare item number.	26.1% of random sample of GPs Features of GPs stated [49].
Britt Australia 2005 [27]	To measure effect on consultation length of GP, practice & patient characteristics.	Jan 2001–Dec 2002	-/1904	70758	14.6 mins [54]	Recorded by doctor	Quantitative analysis of data from BEACH study	21.6% of a random sample of GPs. Features of GPs stated [47]
Zantigne The Netherlands 2005 [33]	To investigate whether GPs' workload in consultations is related to psychological or social problems of patients	2000–20002	-/142	1392 consult'ns	9.06–12.65 mins	Videotaped	Quantitative analysis of data from Second Dutch National Survey of General practice	73% of GPs 88.1% of patients 66% consultations
Wright [35]2005 Australia	To study needs of rural GPs, esp in care of depressed patients	-	-/99. 63 male, 36 fem	-	-	-	Quantitative GP sent postal survey	55%

### Study size

Sample sizes ranged from one where Westcott [25] studied himself, to Britt et al [17]who looked at evidence from over 100,000 consultations with 2,811 doctors.

### Selection of participants

The majority of papers used non-random selection methods, usually volunteers, though six, Howie et al [26], Britt et al [17,27] Rost et al [28], Whitehouse [29] and Blumenthal et al [30] state that they selected a random sample of doctors. Three authors selected the sample of doctors to be representative [31-33] and three invited all the doctors in a particular geographic area [24,34-36]. Whitehouse reported on data from 40% of practices in five health districts in the UK [29]. Some used clearly non-representative samples, for example, Westcott [25] studied one doctor, himself, Andersson et al [37] obtained data from 6 male doctors, all of whom were involved in research, and Stirling et al [9] only included doctors accredited for training.

16 authors reported the participation rates in their papers (see Tables 1, 2 &3), some described the features of the doctors or included practices [9,17,27,28,30,34,38-42]. Four authors compared the features of the participating sample to the non-participating [26,29,35,43]. For example, Deveugle et al [43] and Howe [44] compared the features of those patients and doctors that participated to a representative sample. Deveugle et al reported that doctors involved in the study had a lower workload were more likely to be female and based in a city practice. Zantinge et al stated their sample was "representative for the Dutch population of GPs with regard to age, sex, education, length of residence, degree of urbanisation and number of working hours" [33].

**Table 3:** Relevant data and conclusions of cross-sectional studies on consultation length and management of psychological problems.

**Author**	**Analysis/key data**	**Conclusions/findings**
Westcott 1977 [25]	Psychoneurotic conditions were significantly assoc with longer consultations longer than the median (p < 0.001) mean = 14.14 mins(5–32) Shorter consultations for age group 15–29 years and for lower socioeconomic class.	Psychoneurotic consultations are associated with longer consultations
Raynes 1980 [38]	GPs with positive orientation to mental health spent longer with patients (p < 0.05). Focus on psychosocial matters (p < 0.01), diagnosis of psychological problem & prescription of psychotropic drug resulted in longer consultation	Diagnosis and management of psychological disorder took longer.
Hughes 1983 [3]	Comparison of results between faster & slower practice. Practice A (mean = 8 mins) 7.5% psych diagnosis. Practice B(mean = 5 mins), 7.1%.	No significant difference in psychological problems managed
Whitehouse 1987 [29]	In consultations <6 minutes, 60%of doctors recorded less than 6.3%rate of psychosocial diagnosis. For consultations >8 mins, 34% of doctors recorded over 10% rate., p,0.05, df = 12, x2 = 25	Increasing consultation time assoc with increased diagnosis of psychosocial illness.
Andersson 1989 [37]	Consultations with psychological problems were longer than those with physical, (mean 28 vs 14 minutes).	Consultation for psychological problem took longer compared to physical
Howie 1991 [36]/1989 [24]	Increased cons length assoc with greater recognition & management of chronic illness & psychosocial problems P < 0.05. "Faster" doctors were less likely to deal with a psychosocial problem in depth, when detected p < 0.09	Increased consultation length associated with increase chance of GP dealing with detected psychosocial problem. Longer consultations assoc with reduced prescribing.
Andersson 1993 [52]	The "doctors speed" contributed to 22.5%, the character of the problem 11.6%, the age of the patient 2.9% and the patients sex 0.4%, with coefficient of determination R2 = 0.374. Majority (41% according to dr, 69% according to patient) of short consultations are entirely physical.	The consultation length mainly associated with the doctors "speed" and patient factors including psychological problem and age.
Rost 1994 USA [28]	30% of primary care physicians state that lack of time, & 23% that patient not recognising problem, is the biggest barrier to treating depression	30% of primary care physicians state that lack of time is the biggest barrier to treating depression.
Winefield 1996 [40]	Consultations in top quartile of Patient centerness, compared to bottom quartile, lasted longer (p < 0.001), dealt with more psychosocial or complex problems, had more pt satisfaction (p < 0.05) and same dr satisfaction (p < 0.05), x2 (2d.f.) = 28.84	Patient centred consultations are likely to be longer and include psychological or complex problems.
Martin 1997 [45]	Longer consultations more likely than standard consultations to deal with psychological problems (OR, 2.06; 95%CI 1.83–2.32)	Longer consultations more likely to deal with psychological problems
Carr-Hill 1998 [39]	Multilevel modelling used to analyse assoc of consultation length and multiple factors including diagnosis, doctor, age & gender, & patient age & gender. Average consultation for ICD VIII (Ears) = 5.0 mins, ICD V (Mental & behavioural) = 8.9 mins. ICD XX (social) = 11.8. Only pregnancy longer	Length of consultation explained by variability amongst patients, the diagnosis, GPs & practices. Consultation length not a marker of quality
Howie 1999 [26]	Using multiple regression with enablement as outcome variable, the enablement score was most closely linked to duration of consultations and patient knowing doctor well. Duration of consultation increased for patients with psychological (8.9 mins) or complex problems(9.2 mins) compared to biomedical (7.6 mins).95%CI	Consultation length significant predictor of enablement. Longer consultations for psychological problems(mean = 9.0)
Blumenthal 1999 [30]	Multivariate analysis determined that Psychosocial diagnosis is associated with 9 (6–12)% increase in visit duration, P = <0.001, Age>70, assoc with>11% increase, p < 0.001.	Patient characteristics of increasing age & psychosocial problem are associated with increased duration.
Stirling 2001 [9]	50%increase in consultation length assoc with 32% increase in recognition of psychological distress (95%CI = 10.7–57.3%)	Accurate rating of psychological distress increased with consultation length.
Harman 2001 [42]	Multivariate analysis showing that visits where depression is diagnosed are 2.9 minutes longer on average, 19.3 minutes compared to 16.4 minutes.	Visits with a diagnosis of depression were longer than those without.
Deveugele 2002 [43]	Multilevel analysis with length of consultation a dependent variable. The regression coefficient for diagnosis of psychological problem by the doctor = 0.05(0.08–1.81), for consultation where pt recorded psychosocial aspect important+0.52 (.10–.95)	Increased consultation length associated with positive orientation of doctors to psychosocial problems (not gender); new problems; psychosocial problems perceived by doctor; women patients.
Telford 2002 [34]		GPs believe that time and lack of services are the main obstacles to managing depression, not knowledge or skills.
Tahepold, 2003 [41]	Longest consultation for psychological problem, mean = 11+/- 5.0 mins, p < 0.015.	Older patients and those with psychological problems tend to have longer consultations
Britt 2004 [17]	Psychological problems: 6.7%(6.4–6.4) of consultations<20 mins, 11.6%(11.0–12.2) of longer consultations> 20 mins (95%CI)	Psychological, social & female genital problems more frequently managed in longer consultations. Female doctors have longer consultations.
Britt 2005 [27]	Regression coefficient for Psychological problem = +1.75 mins(1.32–2.18), p < 0.001	Variables with positive effect on consultation length include: Female GP, social, psychological or female genital problem & Chronic disease.
Zantigne 2005 [33]	Consultations with psychological problems are longer than those for somatic problems.12.65 mins compared to 9.06, p < 0.01	Consultations where a GP notices psychological problems make heavier demands on the GPs' workload
Wright 2005 [35]	Ranking of 1–5. Time constraints main barrier to providing care for depressed patients; ranking = 3.04(0.92)	The most common barrier to providing care for depressed patients was "time constraints"

**Table 4:** Intervention studies assessing the effect of booking interval on psychological diagnosis

**Author/year/location**	**Aim**	**No. of doctors/practices**	**No of patients/consultations**	**Mean consultation length**	**Method of measuring consultation length**	**Method of study**	**% of eligible participating**	**Analysis**	**Conclusions/findings**
Morrell 1986 [50] Roland 1986 [53] UK	To measure variables in relation to consultations booked at different intervals	5/1	60 sessions 780 consultations	Booked at 5 mins-mean actual time 5.2; 7.5 mins- 6.7 and 10 mins-7.4 mins.	Booked intervals & Actual time measured on audiotape	Patients non-systematically booked in at varying intervals. Consultations audio taped and analysed. Dr completed encounter sheet Patient questionnaire	80% 20% incomplete	Logistic regression analysis.	Psychological diagnosis more likely to be recorded in consultations booked at longer intervals. Longer consultations associated with more time being spent on history taking.
Risdale 1989 UK [51]	To study the effect of different appointment intervals on process and outcome measures in GP consultations	2/1	961	Booked at 5 mins-mean 6.6;Booked at 10 mins-mean 8;Booked at 15 mins-mean 9.2	Visits audio taped.	Intervention Consultations audio taped and analysed, using same techniques as Morrell and Roland Patient questionnaires	96% of pts agreed to participate. Data complete for 95% of consultations	Regression analysis of various outcome variables.	Increased consultation length assoc with increased doctor questions, patient questions & statements.

Nine papers were part of larger studies [17,27,29,30,33,39,42,43,45] and in four cases [17,27,42,45] the method of selection was obtained from earlier articles or reports [46-49].

### Method of data collection

A variety of methods were used for data collection, ranging from self-completed postal surveys, face to face interviews to direct observational methods that were analysed by independent researchers. In three studies GPs were sent surveys through the post [34,35,44] and in four studies semi-structured or in-depth interviews were conducted with GPs [28,31,32,44]. All the studies based on interviews involved smaller numbers of doctors compared to numbers involved in the postal surveys. Ten of the studies used doctor encounter forms or questionnaires, that were completed at the time of the consultation, eight used patient questionnaires [9,26,36-38,50-52] and in five of these both doctor and patients questionnaires or encounter forms [9,24,26,36,37,52] were used to collect data.

Three studies analysed audiotaped recordings of the consultations [40,50,51,53], three used videotape recordings [33,41,43] and in two studies there were independent observers who recorded and then analysed the consultations [38,39]. Two Australian studies used Medicare billing data [17,45]. Nine studies used data from larger surveys, for example, Britt et al used data from the BEACH survey (Bettering the Evaluation and Care of Health) [17,27], Blumenthal et al and Harman et al used data from the National Ambulatory Medical Care Survey [30,42,46], Zantinge et al from the Second Dutch National Survey of General Practice [33] and Deuvegele et al from the Euro-communication study.

All of these larger studies used data recorded by the doctor [17,27,29,30,33,39,42,43,45].

### Estimation of consultation length

Tables 1, 2 and 4 contain information on the method used to measure consultation length for each study. The most accurate method was the recording of the consultations with audiotaping [40,50,51] or videotaping [33,41,43] or use of an independent observer who sat in the same room [38,39]. In ten cases the consultation length was recorded by the doctor [17,24-27,29,36,37,42,52]. The least accurate method was estimation of consultation length based on the booking interval [31,32]. Four studies did not include a measurement of consultation length but reported on the views of the GPs [28,34,35,44]. In two Australian studies Medicare data on billing were used [17,45]. In these studies the estimate of mean consultation length was obtained from a government report [54].

### Diagnosis of psychological problems

In 12 studies the diagnosis of a psychological problem was made by the doctor during the consultation and recorded by the doctor [3,9,17,24,25,27,29,30,33,36,38,50,51], in one study by both the doctor and by patient questionnaire [9]. Only Stirling at al used a recognised screening tool, the General Health Questionnaire-12 (GHQ), to assess accuracy of the GPs psychological diagnosis. In two studies the diagnosis was made by an independent observer by analysis of videotapes [41,43].

### Time frame

All studies included data on individual consultations only. None of the studies recorded any data over time in order to follow outcomes.

### Outcome measurements

Most studies used study-specific questionnaires with only a few using a recognised outcome measure. Howie et al [26] used the "patient enablement" instrument and Winefield et al [40] explored two previously used measures of "patient-centredness". Stirling et al [9] used the GHQ.

## Data synthesis

Comparing and contrasting study findings yielded three major themes:

1. Consultations with a recorded diagnosis of psychological problems take longer than consultations without a recorded psychological diagnosis.

2. GPs report that time pressure was one of the main barriers to addressing psychosocial problems.

3. There was some evidence of improved outcomes with longer consultations in the diagnosis of psychological problems.

### 1. The length of consultation with a recorded diagnosis of psychological problems

Although the studies report different mean lengths of general practice consultations and different methods of measuring consultation length, 14 out of 16 included studies suggest that consultations with a recorded diagnosis of psychological problems take longer.

These results come from studies conducted in a number of countries, including the UK [25,38,39], the USA [30], Sweden [37], Australia [27,45] The Netherlands [33] and Estonia [41]. This is despite there being a wide variation in the mean lengths of general practice consultations in these countries. Germany and Spain have a mean consultation length of between seven and eight minutes [43], Estonia nine minutes [41], UK 9.4 minutes [43], Netherlands 10.2 minutes [43] and Australia 14.6 minutes [54]. Belgium, Switzerland [43] and USA [30] have average consultation lengths between 15 and 20 minutes, and Sweden averages more than 20 minutes.

The studies by Britt et al [17,27], Deveugele et al [43], Blumenthal et al [30], Zantinge et al [33]and Howie et al [24,26,36] all reported that consultations with a recorded diagnosis of psychological problems take longer. These were large studies with between 1000 to over 100,000 patients, and between 100 to over 1000 doctors. These studies all used rigorous methods including either random selection of participants, use of a representative sample, or description of the participants and/or comparison with non-participants, and analysis techniques involving multilevel or regression analysis.

Deveugele et al compared the length of videotaped consultations across six European countries (Belgium, UK, The Netherlands, Switzerland, Spain, Germany) [43]. They found that the type of patient and the recorded problem largely determined the consultation length. They reported that consultations for patients with a diagnosis of psychosocial problems lasted longer than those with only "biomedical problems".

Zantinge et al explored factors related to the workload of GPs and found that consultations with a psychological diagnosis took longer and also that they were associated with a higher mean number of diagnoses and higher assessment (by the doctors) of insufficient time [33].

Even Carr-Hill, who was attempting to disprove the association between consultation length and quality, showed a definite increase in consultation length in consultations with a diagnosis of psychological problems compared to other consultations [39].

Only two studies, Hughes[3] in 1983 and Ridsdale et al [51] in 1989, did not find that the length of booked appointments effected the rate of management of psychological problems. However, these studies only studied one or two practices, Hughes did not individually time the consultations [3] and they both reported a rate of 8% or less of diagnosis of psychological problems. Ridsdale et al suggested that the use of small cards used as the medical record in the practices studied inhibited the recording of notes and hence reduced the rate of recording of psychological problems [51].

Otherwise, all the studies concur with the conclusion that consultations with a recorded diagnosis of psychological problems did take longer.

Many of the studies explored possible confounding factors that may influence consultation length. These included consultation style, doctors' attitudes and other "doctor" factors and "patient" factors.

#### Consultation length and doctors' style

Six [24,26,36,40,50,51,53] studies explored confounding factors during the consultation that could explain the increase in diagnosis of psychological problems by documenting changes in consultation style with variation in time. Two of these were intervention studies which explored the effect of alteration of the booking interval and hence the time available to the doctor. Morrell et al [50] and Roland et al [53] reported on the same study which demonstrated that increasing the booking interval resulted in longer consultations and greater diagnosis of psychological problems. They also reported a change in communication style in longer consultations, resulting in the physician spending more time talking and listening to the patients, and more psychosocial questions being asked.

Ridsdale et al did a similar study and their results demonstrated that in the longer consultations there was a change in consultation style, or communication patterns, with more doctor questions and explanations, and more patient questions and statements. However, longer consultations did not lead to more psychological diagnoses recorded in the notes [51].

The other four studies exploring consultation style were observational, with no intervention. Howie et al have documented an evolving study of consultation styles and the effect on the consultation length while exploring issues of quality of care [24,26,36]. Using observational data Howie et al found that doctors could be grouped as "fast", "intermediate" and "slow". They found that faster doctors were less likely to deal with psychosocial problems and more likely to prescribe [24]. They repeated a similar study in 1991 to explore in more depth the differences between "faster" and "slower" doctors and found that "slower" doctors deal with more psychological problems even when they saw patients in shorter consultations [36].

Rather than focus on consultation time alone, some researches have set about to explore styles within the consultation. In 1999, Howie et al used the concept of "patient enablement" and found that consultations with a psychological diagnosis tended to take longer and to be associated with a higher enablement score, as measured by the Patient Enablement instrument. They found that even in short consultations, doctors who were high enablers continued to have a higher enablement score than low enablers. [26].

This evidence is supported by Winefield et al whom assessed the association between consultation length, patient centredness and psychological diagnosis. They reported that consultations in the top quartile for patient-centredness, compared to the bottom quartile, lasted longer and dealt with more psychosocial and complex problems [40].

#### Consultation length and doctor factors

Three studies explored the doctors' attitude to psychological problems to explain the connection between longer consultations and the increased diagnosis of psychological problems. Deveugele et al [43] and Raynes et al [38] reported that doctors who tend to have longer consultations are more likely to have a positive attitude to psychological problems. Similarly, Howe concluded in her paper that "a GP who adopts a more open consulting style is more likely to improve their performance as a detector of psychological distress" [44].

"Doctor" factors, including age, gender and training, which could be confounding factors for longer consultations were documented in eight studies. Four studies looked at doctor age and gender and its effect on consultation length. Two demonstrated that female doctors tend to have longer consultations [27,39] and two did not show this difference [29,43]. Britt et al [27] showed an increased duration of consultation with increasing doctor age but this was not consistent in other studies [29,43,52]. Two studies showed an increase in consultation length with increased doctor training [27,45], while three [29,39,43] did not find this difference. There was an expected finding of an increased length of consultations with an increased number of (or with more than one) diagnoses [17,27,33,39,41,52].

#### Consultation length and patient factors

Thirteen of the studies explored "patient" factors associated with longer consultations. Six out of seven studies demonstrated that there was increased consultation length with increasing patient age [27,30,37,41,43,52] and one reported changes with patient age that were not linear [38].

Five out of seven studies reported that women patients were found to have longer consultations [17,27,37,43,52], with Deveugele et al noting that the longest consultations were women with psychosocial problems [43], and Carr-Hill et al reporting that middle-aged women had the longest consultations [39]. Two studies did not find this difference [38,41]. Whitehouse reported that women patients have twice the number of consultations with psychosocial diagnoses [29].

Four out of five studies reported on the association between consultation length and educational level, or socioeconomic status, of the patient. One [45] reported that patients who were well educated were more likely to have longer consultations, three reported that patients with higher SES generally had longer consultations [9,25,27] and one concluded that there was no difference [43].

There were four other reported confounding "patient factors" that were related to a change in the consultation length. Firstly, that "new patients" were associated with longer consultations [17,27,43] and, secondly, that continuity of care (that is, more than one visit with the same doctor) was associated with shorter consultations [26,37,52]. Thirdly and not surprisingly, patients presenting with more than one problem were associated with having longer consultations [43,45].

Finally, two studies combined doctor and patient factors and reported that female doctors treating female patients have the longest consultations [27,39].

### 2. GPs views on barriers to treating psychological problems

Six papers reported results from surveys and interviews with doctors' about their views on the management of psychological problems, particularly depression. Four were qualitative studies and two quantitative [28,31,32,34,35,44].

Four of these studies reported that GPs' believed that time was the major barrier to the management of psychological problems [28,32,34,35]. Three studies explored other possible barriers to the treatment of depression and reported that access to necessary services and resources, not issues with GPs knowledge or skills, were major obstacles [28,32,34]. Pollock et al explored GPs views on the consultation length for patients with depression and reported that GPs believe that consultations for depression were often longer, especially the first consultation, and that GPs accommodated patients with depression by running over time [31]. Rost et al also reported that patient factors, particularly the failure of the patient to recognise their depression, were a barrier [28].

Howe reports that GPs believe that they have the necessary skills but that the lack of time is the biggest barrier to effective detection of depression during a consultation[44]. Five of the GPs in her study "mentioned the likelihood of rating patients as not distressed if they themselves could not face dealing with that aspect of care."

### 3. Evidence of improved diagnosis of psychological problems with longer consultations

One study used an outcome measurement in the diagnosis of psychological problems. Stirling et al [9] examined factors, including consultation length, associated with accuracy of diagnosis of psychosocial distress in general practice. They measured accuracy by comparing the doctors rating of psychological distress with a GHQ (General Health Questionnaire-12) score completed by the patient. The accurate recognition of psychological distress was greater in longer consultations, with a 50% increase in consultation length being associated with a 32% increase in recognition. They also compared the rating of psychological distress with social deprivation and consultation length, finding that greater socioeconomic deprivation was associated with greater GHQ scores, but that increased social deprivation was associated with shorter consultations.

## Discussion

When undertaking this review we deliberately used broad inclusion criteria in an effort to scope the evidence relating to consultation length and management of psychological problems. We initially hoped to answer the important question: "Do longer consultations result in improved outcomes in the management of psychological problems in general practice?" Unfortunately, there was insufficient data to answer this question conclusively and there were a number of important limitations in studies identified.

We identified 29 relevant papers from a mix of western countries, but, despite the consultation being a crucial component of general practice work, all studies reviewed were observational and non-randomised studies. We were unable to identify a single randomised controlled trial where the consultation length had been altered to measure effects on the diagnosis or the management of psychological problems.

Apart from one paper by Stirling et al [9], on accuracy of psychological diagnosis, there were no studies found that explored association between outcome measurements or management options of psychological problems and length of consultation.

Another major weakness is that none of the included studies followed patients to assess progress, all studies involved single visits.

Despite this, there were three possible conclusions. The strength of evidence for these conclusions varies and will be discussed.

### 1. Consultations with a recorded diagnosis of psychological problems take longer

Despite the mean consultation length varying between countries, evidence from 14 studies from 10 different countries, with a wide range of health systems, payment systems, cultural backgrounds, doctor demand and training demonstrated that consultations with a recorded diagnosis of psychological problems take longer than those without. The studies supporting this conclusion varied in size and methods used. The studies included large and small studies, representative and non-representative samples, consultations were timed in a variety of ways and the diagnosis of psychological problems was made sometimes by the doctor, sometimes by the patient and sometimes by an independent observer.

There were a number of possible confounding factors that may have resulted in changes to the number of recorded diagnoses of psychological problems between longer consultations and "average" consultations.

It may be that in longer consultations, doctors had more time to record more thorough notes.

Although this is a possible explanation, it is disputed by the studies that included patient questionnaires [9], and where the diagnosis of psychological problems was made by independent observers from videotapes [41,43].

If we accept that the increase in recorded diagnoses of psychological problems was a real increase, the issue then arises as to whether the consultations took longer because of the diagnosis of a psychological problem, or if the diagnosis was made more often by doctors who tended to have longer consultations? This question is explored further using evidence from this review on the possible effects of the consultation styles, doctor attributes and patient attributes on the length of consultation.

#### Consultation length and doctors' style

We found evidence from five relevant studies to support the hypothesis that doctors with consultation styles that took more time were more likely to make a psychological diagnosis. Several "doctor styles" were explored and all these styles result in more psychological diagnoses and took more time.

As well, the evidence from the two intervention studies clearly demonstrated changes in consultation style when the doctor had more time [50,51]. Unfortunately, the intervention studies had limitations as both studies only collected data from one practice each, one [50] in the inner city and the other [51] in a suburban practice. Also, no information was given on the attitudes, ages or styles of the doctors involved in either study.

These studies raise the interesting question about whether it is the time or the doctors' style that was most important in diagnosing psychological problems. More evidence, including randomised controlled trials, is necessary to clarify this hypothesis.

#### Consultation length and doctor factors

We looked for evidence that reported on doctors' attributes apart from consultation style that may increase the likelihood of a diagnosis of psychological problems and of longer consultations. Two of these studies highlighted the importance of a positive attitude to psychological problems [38,43] on the diagnosis rate and Howe commented that doctors sometimes make a "choice" about whether to diagnose and manage the psychological problems presenting in a consultation depending on their "time and energy" [44]. This raises the question as to whether this attitude is part of the doctor's character or due to training, and hence whether the selection criteria for medicine and the training are appropriate. It also raised the likely effect of time pressure on a doctors' ability to offer optimal care.

Evidence was presented on other "doctor attributes" that are associated with longer consultations, particularly doctors' age, gender and training. However, this evidence is conflicting and does not clearly demonstrate confounding factors.

#### Consultation lengths and patient factors

We then looked for data on the patient attributes, apart from psychological problems, which may be associated with an increase in the length of consultations. It appears that increasing patient age is associated with longer consultations [27,30,37,38,41,43,52]. This is important information for health policy-makers who are dealing with ageing populations.

We found that five out of seven studies reported that women patients had longer consultations [17,27,37,38,41,43,52], especially women patients with psychosocial problems [43] and that women had twice number of consultations with psychosocial diagnoses [29]. This raises the question about why women have longer consultations, could they be better at expressing their psychological distress, or do they actually have a higher rate of psychological distress?

There appeared to be an association between increased socioeconomic deprivation and greater psychological distress, but also with shorter consultations[9]. This "inverse care law" has been further examined by Furler et al who demonstrated that patients in "lower socioeconomic areas receive less longer consultations than those in more advantaged areas" [55].

While it is clear that an increased recording of psychological diagnosis is associated with longer consultations, more research needs to be done to elucidate how these various factors can be explained and to determine the factors that would enable the most time-efficient, most accurate, method of diagnosis of psychological problems.

### 2.GPs report that time was one of the main barriers to addressing psychosocial problems

The evidence is obtained from six observational, cross-sectional studies [28,31,32,34,35,44]. The numbers varied from 11 to 1700 doctors but only one study stated that it was representative and none of the studies had analysis involving confidence intervals. Hence, this evidence can only be hypothesis forming.

Despite these weaknesses, the evidence suggests that general practitioners consider it is lack of time, and not lack of knowledge, that is preventing them from achieving better outcomes for psychological problems. Howe raises the interesting question of the doctor's choice in the consultation, about whether to pursue the psychological aspects of a consultation depending on other factors, "time and energy" [44]. As Howe put it, GPs "know what to do, but it's not possible to do it." This choice is also reflected in the study by Howie et al where "slower doctors" could work faster if they were under time pressure [26].

This is important information for health policy makers to consider as often it is a perceived lack of skills in general practice that is blamed for deficiencies in the management of psychological illness.

It does, however, contradict other research that shows that GPs will considerably improve the rate of detection of psychological distress with simple training[56,57]. It is likely that both training and time are important.

### 3. There was evidence of improved diagnosis of psychological problems in longer consultations

Stirling [9] provides the only evidence on containing consultation outcomes, in his study on the accuracy of detection of psychological distress; however, there is no reliable evidence that taking longer is related to improved outcomes in management of psychological problems. It is obvious that more research needs to be done in this area.

## Conclusion

From this systematic literature review we can conclude that consultations with a recorded diagnosis of a psychological problem take longer than those with no recorded psychological diagnosis. It is not clear if this is related to the extra time or the consultation style, or other confounding factors. Research is needed to elucidate the factors in longer consultations that are associated with greater detection of psychological problems and to determine whether these are effected by the GPs' attitude, gender, age or training or the patients' age, gender and socioeconomic status. These are important considerations if general practice is to deal more effectively with people with psychological problems.

## Competing interests

The author(s) declare that they have no competing interests.

## Authors' contributions

CH and JG developed the question, CH performed the literature search, retrieved the papers, performed the data extraction, developed a thematic analysis and wrote the first draft of this paper, JG and CH refined the themes and both authors contributed to subsequent drafts and approved the final version.

**Figure 1:**
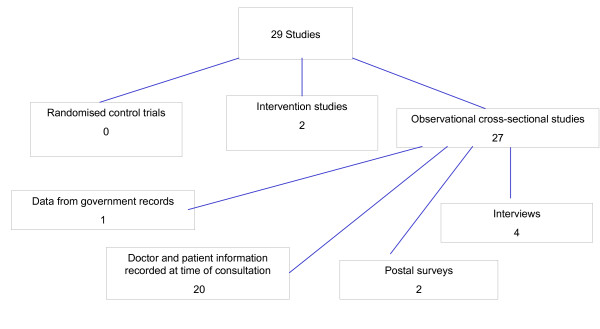
Number of included studies and the study types.

## Pre-publication history

The pre-publication history for this paper can be accessed here:



## References

[B1] Stott NCH, Davis RH (1979). The exceptional potential in each primary care consultation.. Journal of Royal College of General practitioners.

[B2] Wilson A (1991). Consultation length in general practice: a review.. British Journal of General Practice.

[B3] Hughes D (1983). Consultation length and outcome in two group general practices.. Journal of the Royal College of General Practitioners.

[B4] Buchan IC, Richardson IM (1973). Time Study of Consultations in General Practice, Scottish health Service statistics no 27..

[B5] AP H, Freeman GK, Horder JP, Howie JGR, Hungin AP, Shah NC, Wilson A (2002). Evolving general practice consultation in Britain: issues of length and context.. British Medical Journal.

[B6] Australian Institute of Health and Welfare (2005). Chronic Diseases..

[B7] Department of Health and Ageing Commonwealth of Australia (2005). National Mental Health Report 2005: Summary of Ten Years of Reform in Australia's Mental Healthservices under the National Mental Health Strategy 1993-2003..

[B8] Harrison CM, Britt H (2004). The rates and management of psychological problems in Australian general practice.. Australian & New Zealand Journal of Psychiatry.

[B9] Stirling AM, Wilson P, McConnachie A (2001). Deprivation, psychological distress, and consultation length in general practice.. British Journal of General Practice.

[B10] Groom G, Hickie I, Davenport T (2003). Out of Hospital, Out of Mind. A report detailing Mental Health Services in Australia in 2002 and Community Priorities for National Mental Health Policy for 2003-2008..

[B11] Hickie IB, Davenport TA, Scott EM, Hadzi-Pavlovic D, Naismith SL, Koschera A (2001). Unmet need for recognition of common mental disorders in Australian general practice.. Medical Journal of Australia.

[B12] Australian Medical Workforce Advisory Committee (2005). The general practice Workforce in Australia: Supply and requiremants to 2013, AMWAC Report 2005.2,Sydney..

[B13] British Medical Association (2006). Health Committee Inquiry into Workforce Planning,
British Medical Association Memorandum of Evidence..

[B14] Alexander, Wegner (2004). Health Care Industry HGJTI Narrative Report
Health Care Industry Identifying and Adddressing Workforce Challenges..

[B15] Association of American Medical Colleges (2005). The Physician Workforce: Position Statement..

[B16] Medical Council of Canada (2006). Medical Workforce Policy-Making in Canada: 1993-2003. Canada’s Medical Workforce Policy Makers’ Current Challenges. Two Responses to the Enrollment Cutbacks of the 1990.: feb06; Montreal, Canada...

[B17] Britt H, Valenti L, Miller GC, Farmer J (2004). Determinants of GP billing in Australia: content and time.. Medical Journal of Australia.

[B18] Agence France-Presse (2004). China faces serious shortage of medical workers.. http://wwwafpcom/.

[B19] UN News Centre (2005). New UN-backed alliance seeks to reverse worldwide doctor, health worker shortage.. http://wwwunorg/News/ accessed 22 July 2006.

[B20] Mullan F (2005). The Metrics of the Physician Brain Drain.. The New England Journal of Medicine http://contentnejmorg/.

[B21] Groenewegen P, Hutten JBF (1991). Workload and Job Satisfaction among General Practitioners: A Review of the literature.. Soc Sci Med.

[B22] Dugdale DC, Epstein R, Pantilat SZ (1999). Time and the patient-physician relationship.. Journal of General Internal Medicine.

[B23] Wilson A, Childs S (2002). The relationship between consultation length, process and outcomes in general practice: a systematic review.. British Journal of General Practice.

[B24] Howie JG, Porter AM, Forbes JF (1989). Quality and the use of time in general practice: widening the discussion.. BMJ.

[B25] Westcott R (1977). The length of consultation in General Practice.. JR Coll Gen pract.

[B26] Howie JG, Heaney DJ, Maxwell M, Walker JJ, Freeman GK, Rai H (1999). Quality at general practice consultations: cross sectional survey.. British Medical Journal.

[B27] Britt H, Valenti L, Miller G (2005). Determinants of consultation length in Australian general practice.. Medical Journal of Australia.

[B28] Rost K, Humphrey J, Kelleher K (1994). Physician management preferences and barriers to care for rural patients with depression.. Arch Fam Med.

[B29] Whitehouse C (1987). A survey of the management of psychosocial illness in general practice in Manchester.. Journal of the Royal College of General Practitioners.

[B30] B. S, Blumenthal D, Causino N, Chang YC, Culpepper L, Marder W, Saglam D, Stafford R (1999). The duration of ambulatory visits to physicians.. J Fam Pract.

[B31] Pollock K, Grime J (2003). GPs' perspectives on managing time in consultations with patients suffering from depression: a qualitative study.. Family Practice.

[B32] Smith L, Walker A, Gilhooly K (2004). Clinical guidelines of depression: a qualitative study of GPs' views.. Journal of Family Practice.

[B33] J. B, Zantinge EM, Verhaak PF, Kerssens JJ (2005). The workload of GPs: consultations of patients with psychological and somatic problems compared.. British Journal of General Practice.

[B34] Telford R, Hutchinson A, Jones R, Rix S, Howe A (2002). Obstacles to effective treatment of depression: a general practice perspective.. Family Practice.

[B35] Wright MJ, Harmon KD, Bowman JA, Lewin TJ, Carr VJ (2005). Caring for depressed patients in rural communities: general practitioners' attitudes, needs and relationships with mental health services.. Aust J Rural Health.

[B36] Howie JGR, Porter AMD, Heaney DJ, Hopton JL (1991). Long to Short Consultation Ratio - a Proxy Measure of Quality of Care for General-Practice.. British Journal of General Practice.

[B37] Andersson SO, Mattsson B (1989). Lengths of consultations in General practice in Sweden-Views of doctors and patients. Family Practice.

[B38] Raynes NV, Cairns V (1980). Factors contributing to the length of general practice consultations.. Journal of the Royal College of General Practitioners.

[B39] Carr-Hill R, Jenkins-Clarke S, Dixon P, Pringle M (1998). Do minutes count? Consultation lengths in general practice.. Journal of Health Services & Research Policy.

[B40] Winefield H, Murrell T, Clifford J, Farmer E (1996). The search for reliable and valid measures of patient-centredness.. Psychology & Health.

[B41] Tahepold H, Maaroos HI, Kalda R, van den Brink-Muinen A (2003). Structure and duration of consultations in Estonian family practice.. Scand J Prim Health Care.

[B42] Harman JS, Schulberg HC, Mulsant BH, Reynolds CF3rd (2001). The effect of patient and visit characteristics on diagnosis of depression in primary care.. J Fam Pract.

[B43] Deveugele M, Derese A, van den Brink-Muinen A, Bensing J, De Maeseneer J (2002). Consultation length in general practice: cross sectional study.. British Medical Journal.

[B44] Howe A (1996). ''I know what to do, but it's not possible to do it'' - General practitioners' perceptions of their ability to detect psychological distress.. Family Practice.

[B45] Martin CM, Attewell RG, Nisa M, McCallum J, Raymond CJ (1997). Characteristics of longer consultations in Australian general practice.. Medical Journal of Australia.

[B46] Woodwell D (2000). National Ambulatory Medical Care Survey: 1998 Summary.. Vital and Health Statistics of the Centres for Disease Control and Prevention.

[B47] Britt H, Miller G, Knox S, Charles J, Valenti L, Pan Y, Bayram C, Henderson J, O'Halloran J, Ng A (2004). General practice activity in Australia 2003-2004. General Practice series No.16..

[B48] McCallum J, Lonergan J, Raymond C (1993). The NCEPH Record Linkage Pilot Study: A prelimnary examination of individual Health Insurance Commission records with linked data sets..

[B49] Britt H, Miller GC, Knox S, Charles J, Valenti L, Henderson J, Pan Y, Bayram C, Harrison C (2003). General practice activity in Australia 2002-2003. General Practice series No.14..

[B50] Morrell DC, Evans ME, Morris RW, Roland MO (1986). The "five minute" consultation: effect of time constraint on clinical content and patient satisfaction.. British Medical Journal Clinical Research Ed.

[B51] Ridsdale L, Carruthers M, Morris R, Ridsdale J (1989). Study of the effect of time availability on the consultation.. Journal of the Royal College of General Practitioners.

[B52] Andersson SO, Ferry S, Mattsson B (1993). Factors associated with consultation length and characteristics of short and long consultations. Scandinavian Journal of Primary Health Care.

[B53] Roland MO, Bartholomew J, Courtenay MJ, Morris RW, Morrell DC (1986). The "five minute" consultation: effect of time constraint on verbal communication.. British Medical Journal Clinical Research Ed.

[B54] Australian Institute of Health Workforce (2002). Consultation time and GP satisfaction, General Practice series No3..

[B55] Furler JS, Harris E, Chondros P, Davies PGP, Harris MF, Young DYL (2002). The inverse care law revisited: impact of disadvantaged location on accessing longer GP consultation times.. Medical Journal of Australia.

[B56] Pawlikowska TRB, Nowak PR, Szumilo-Grzesik W, Walker JJ (2002). Primary care reform: a pilot study to test the evaluative potential of the Patient Enablement Instrument in Poland.. Fam Pract.

[B57] Scott J, Jennings T, Standard S, Ward R, Goldberg D (1999). The impact of training in problem-based interviewing on the detection and management of psychological problems presenting in primary care.. British Journal of General Practice.

